# In Silico Analysis Revealed Marco (SR-A6) and Abca1/2 as Potential Regulators of Lipid Metabolism in M1 Macrophage Hysteresis

**DOI:** 10.3390/ijms26010111

**Published:** 2024-12-26

**Authors:** Yubo Zhang, Wenbo Yang, Yutaro Kumagai, Martin Loza, Yitao Yang, Sung-Joon Park, Kenta Nakai

**Affiliations:** 1 Department of Computational Biology and Medical Science, The University of Tokyo, Tokyo 108-8639, Japan; yzhang951221@gmail.com (Y.Z.); jack903418830@gmail.com (W.Y.); yang-yitao@g.ecc.u-tokyo.ac.jp (Y.Y.); 2 Department of Life Science and Biotechnology, National Institute of Advanced Industrial Science and Technology, Tokyo 305-0044, Japan; yutaro.kumagai@aist.go.jp; 3 Human Genome Center, The Institute of Medical Science, The University of Tokyo, Tokyo 108-8639, Japan; mloza@g.ecc.u-tokyo.ac.jp (M.L.); sjpark@ims.u-tokyo.ac.jp (S.-J.P.)

**Keywords:** macrophage hysteresis, innate immune memory, macrophage polarization, macrophage lipid metabolism, macrophage reprogramming

## Abstract

Macrophages undergo polarization, resulting in distinct phenotypes. These transitions, including de-/repolarization, lead to hysteresis, where cells retain genetic and epigenetic signatures of previous states, influencing macrophage function. We previously identified a set of interferon-stimulated genes (ISGs) associated with high lipid levels in macrophages that exhibited hysteresis following M1 polarization, suggesting potential alterations in lipid metabolism. In this study, we applied weighted gene co-expression network analysis (WGCNA) and conducted comparative analyses on 162 RNA-seq samples from de-/repolarized and lipid-loaded macrophages, followed by functional exploration. Our results demonstrate that during M1 hysteresis, the sustained high expression of Marco (SR-A6) enhances lipid uptake, while the suppression of Abca1/2 reduces lipid efflux, collectively leading to elevated intracellular lipid levels. This accumulation may compensate for reduced cholesterol biosynthesis and provide energy for sustained inflammatory responses and interferon signaling. Our findings elucidate the relationship between M1 hysteresis and lipid metabolism, contributing to understanding the underlying mechanisms of macrophage hysteresis.

## 1. Introduction

In the human body, macrophages perform fundamental functions such as maintaining hemostasis and resisting pathogen invasion [1]. Upon exposure to various environmental stimuli, macrophages polarize into distinct phenotypes within different tissues, including M1 macrophages and M2 macrophages [2]. For example, lipopolysaccharide (LPS) can drive polarization toward the M1 phenotype, while interleukin-4 (IL-4) promotes the polarization of the M2 subtype [3]. M1 macrophages are primarily responsible for triggering inflammatory responses, whereas M2 macrophages serve to balance inflammation and promote wound healing and tissue repair [4]. In inflammatory tissues, macrophages often undergo M1 polarization initially and are subsequently repolarized into the M2 phenotype for tissue repair. This transition from one phenotype to another is referred to as repolarization or reprogramming.

Numerous studies have detailed memory T and B cells [5,6,7], yet the concept of innate memory and specifically memory macrophages was only established about a decade ago. Recent research challenges the traditional view that relegates innate immune cells merely to the first line of defense, suggesting instead that these cells can also develop and maintain immunologic memory or hysteresis [8,9]. Multiple independent clinical and biological studies have confirmed such macrophage hysteresis [10,11,12,13,14]. This de facto innate immune memory, referred to as trained immunity, is mediated through extensive metabolic rewiring and epigenetic modifications [15]. Energy and lipid metabolisms, along with dietary compounds, influence enzymes that regulate chromatin compaction and structure, whereby lipid metabolites play a critical role in mediating epigenetic modifications that impact macrophage immune memory, significantly contributing to trained immunity [16]. Lots of experiential evidence underscores the significant influence of lipid metabolism on innate immune memory and macrophage hysteresis [17,18].

**Figure 1:**
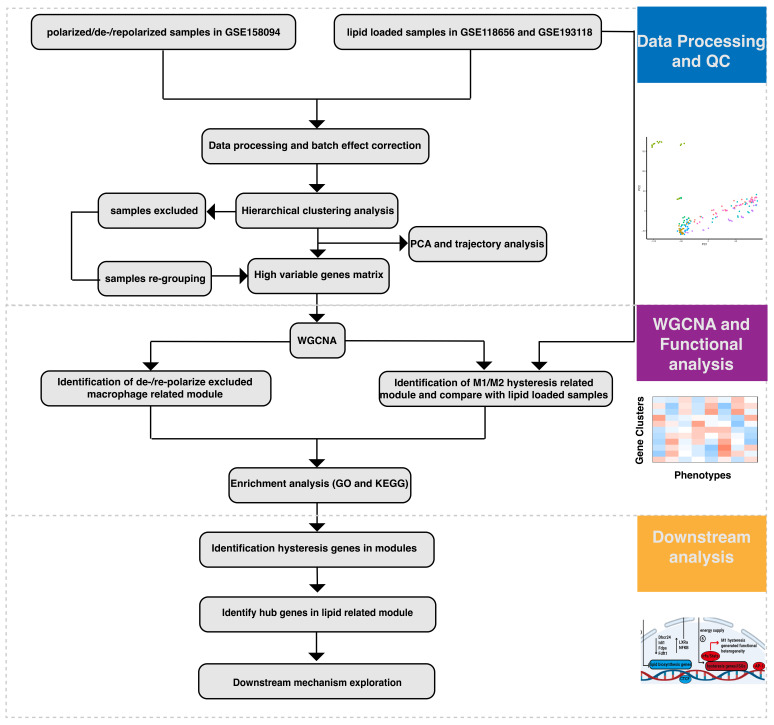
Flowchart illustrating the study design. Workflow for data processing and quality control, weighted gene co-expression network analysis (WGCNA), functional analysis, and downstream analysis in this study.

In our previous research, we reported significant hysteresis in bone marrow-derived macrophages following M1 stimulation and subsequent de-/repolarization, attributed to the regulation of various chromatin sites by factors such as AP-1 and CTCF. This sustained M1 hysteresis induces functional heterogeneity in macrophages, affecting inflammatory responses, cell cycle regulation, and cell migration. Remarkably, we observed a hysteresis phenomenon in a set of interferon-stimulated genes (ISGs) [9]. Research by Lisa Willemsen et al. indicates that lipid accumulation in mouse and human macrophages leads to the differential expression of type I interferons [19]. Furthermore, studies suggest that lipid metabolism is crucial for mounting effective inflammatory responses [20]. We hypothesize that the observed M1 hysteresis may be directly linked to macrophage lipid metabolism. Thus, in the present study, we analyzed a total of 162 RNA-seq datasets from polarized and lipid-loaded macrophages, applying WGCNA to identify gene modules and hub genes strongly associated with macrophage hysteresis. Subsequent mechanistic investigations were conducted to explore the potential correlation between M1 hysteresis and lipid metabolism, as well as their underlying interactions (Figure 1).

## 2. Results

### 2.1. Trajectory Analysis and Hierarchical Clustering Confirmed the High Sensitivity of Macrophages to the Time of Stimulation

**Figure 2:**
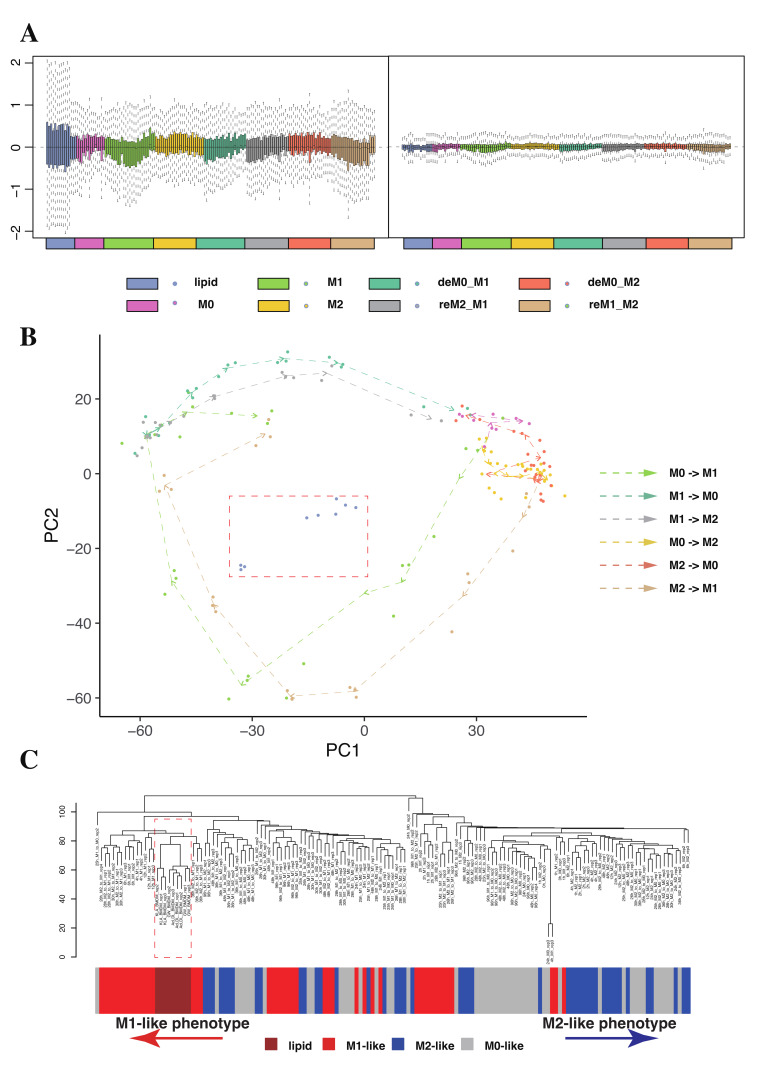
Batch effect correction and quality control in the macrophage gene expression data. (**A**) Relative gene expression levels (Z-score) among the RNA-seq data before and after the batch effect correction and (**B**) trajectory PCA plot of macrophage RNA-seq expression across all phenotypes. The colored dots represent macrophages in different phenotypes and arrows represent polarization trajectory. The points within the red dashed box represent lipid-loaded samples. (**C**) Hierarchical clustering based on RNA expression of all samples. The red arrow indicates the M1-like phenotype direction, while the blue arrow indicates the M2-like direction. Lipid-loaded macrophage samples are enclosed in a red dashed box.

We first performed batch effect correction on the TPM-transformed RNA expression matrix, containing 162 samples, which is described in the Section 4. We compared the deviation in the expression levels before and after batch effect correction (Figure 2A). We observed that after batch effect correction using mouse housekeeping genes, both intra-group and inter-group expression variations were significantly reduced across all samples, indicating the successful elimination of batch effects. To further describe the distribution of all samples and validate sample quality, we compared the PCA plot generated from the original TPM matrix (Figure S1) with the PCA plot after batch removal (Figure 2B). In the PCA plot after batch removal, the trajectory of M1 polarization appeared longer than that of M2 polarization, consistent with previous findings by Liu et al. [21].

However, the lipid-loaded macrophages, highlighted with a red dashed-line box, exhibited a differential expression pattern compared to all samples across the macrophage polarization trajectory, although we observed that lipid-loaded macrophages were closer to the M1 or M1-like phenotypes (M1 and reM1_M2). This difference is not attributable to batch effects and is likely to indirectly affect the selection of gene clusters in subsequent WGCNA, as the overall gene expression levels of lipid-loaded samples may exhibit a relatively low correlation with those of other polarized phenotypes. We then performed a preliminary WGCNA experiment using lipid samples as a phenotype (Figure S2). The results indicated that the lipid phenotype exhibited very low and non-significant correlations with all ten gene clusters compared to the other phenotypes in Figure S2. Therefore, we considered not using the lipid samples directly in the WGCNA but rather employing them in subsequent analyses to validate the significantly expressed lipid metabolism-related genes discovered in polarized samples.

To further ascertain the distribution among the samples, we employed the hierarchical clustering method (Figure 2C). The results confirm that lipid samples marked with a red dashed-line box clustered together and were distinctly different from other polarized samples. Interestingly, the results indicate that the distribution of all samples does not center from the M0-like phenotype and extends towards the M1 and M2 directions, underscoring that in addition to the type of stimulation, the duration of stimulation also significantly influences the characteristics of macrophages (Figure 2C). Based on the analysis above, we have discovered the following: 1. Lipid-loaded samples are not suitable to be directly analyzed as a distinct phenotype alongside other polarized samples as this could significantly impact the analysis results. 2. Due to the high plasticity of macrophages and their extreme sensitivity to the duration of stimulation, polarized macrophages under different stimulation durations require further classification.

### 2.2. Considering the Stimulation Time Factor Significantly Improved the Accuracy of Sample Classification for Downstream Analysis

**Figure 3:**
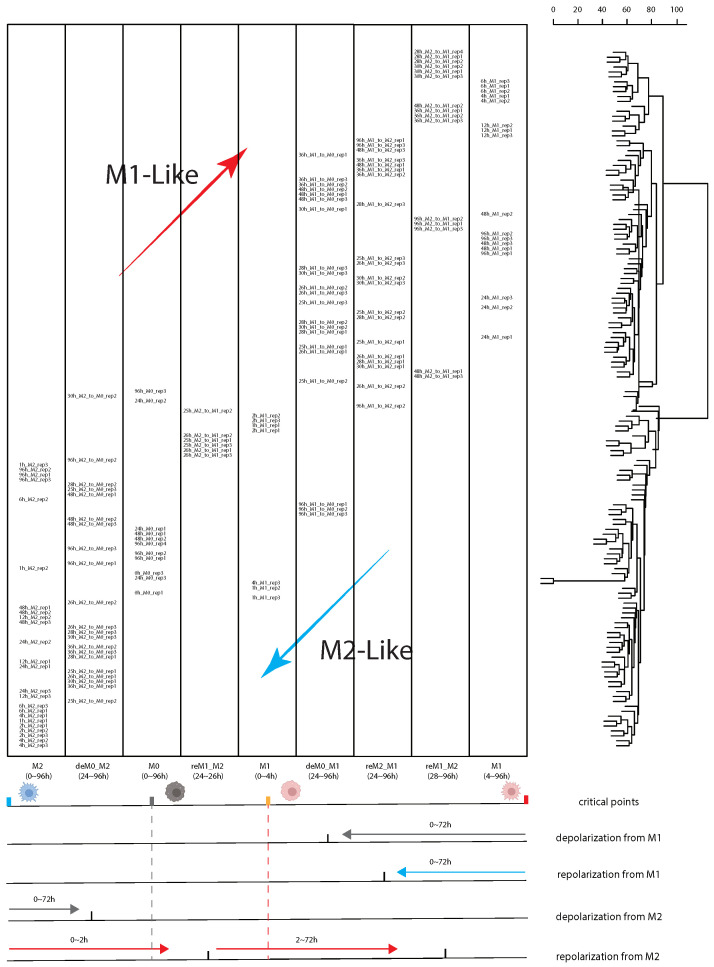
Re-grouping of samples based on macrophage stimulation time. The horizontal axis of the table represents the grouping of macrophages based on different polarization stimulation times and types, while the vertical axis displays the transcriptome distribution tree using hierarchical clustering for all samples. The red and blue arrows in the table indicate trends toward M1-like and M2-like macrophages, respectively. The red arrows below represent M1 repolarization stimulation, the blue arrows represent M2 repolarization stimulation, and the gray arrows represent depolarization. The points on the scale below indicate the approximate positions of each depolarized/repolarized sample, with the M0, M1, and M2 phenotypes serving as critical points.

To accurately classify all the macrophage samples and enhance the significance of the subsequent analyses, we incorporated annotations of stimulation time to the hierarchical clustering tree in Figure 2C (Figure 3). We used the positions of M0, M1, and M2 samples in the hierarchical clustering tree as critical points, which indicate when macrophages are about to change their original phenotypes. The results show that macrophages polarized to M1 from 0 to 4 h exhibit a significant differential expression compared to those polarized for extended periods (4 to 24 h). This explains why, in the previous classification, these M1-polarized macrophages were categorized under the M2-like group. Similarly, M2 macrophages undergoing M1 repolarization for less than 2 h (reM1_M2, 24–26 h) also show substantial differential expression compared to cells stimulated for longer periods (reM1_M2, 28–96 h). Evidently, this is because these M0 and M2 cells have not completed polarization or repolarization within the short time frame, and categorizing these cells as either M1-like or M2-like based solely on polarization type without considering the extent of polarization would severely impact the subsequent analyses. Additionally, we observed that the depolarized M1 cells at 96 h exhibit a state more similar to the M0 phenotype rather than the M1 phenotype, which led us to differentiate these samples from the depolarized M1 cells stimulated for 24 to 48 h.

Based on our analyses and observations, we subsequently re-categorized all samples into the following nine groups for downstream analysis to minimize the impact caused by incomplete cellular polarization or a short period of stimulation: M0, 0~4 h M1 (0_4M1), 4~96 h M1 (4_96M1), M2, 24~26 h repolarized M1 from M2 (24_26reM1_M2), 28~96 h repolarized M1 from M2 (28_96reM1_M2), 24~48 h depolarized M0 from M1 (24_48deM0_M1), 96 h depolarized M0 from M1 (96deM0_M1), depolarized M0 from M2 (deM0_M2), and repolarized M2 from M1 (reM2_M1) (Table S1).

### 2.3. WGCNA Revealed Specific Gene Modules Associated with Macrophage Phenotypes

**Figure 4:**
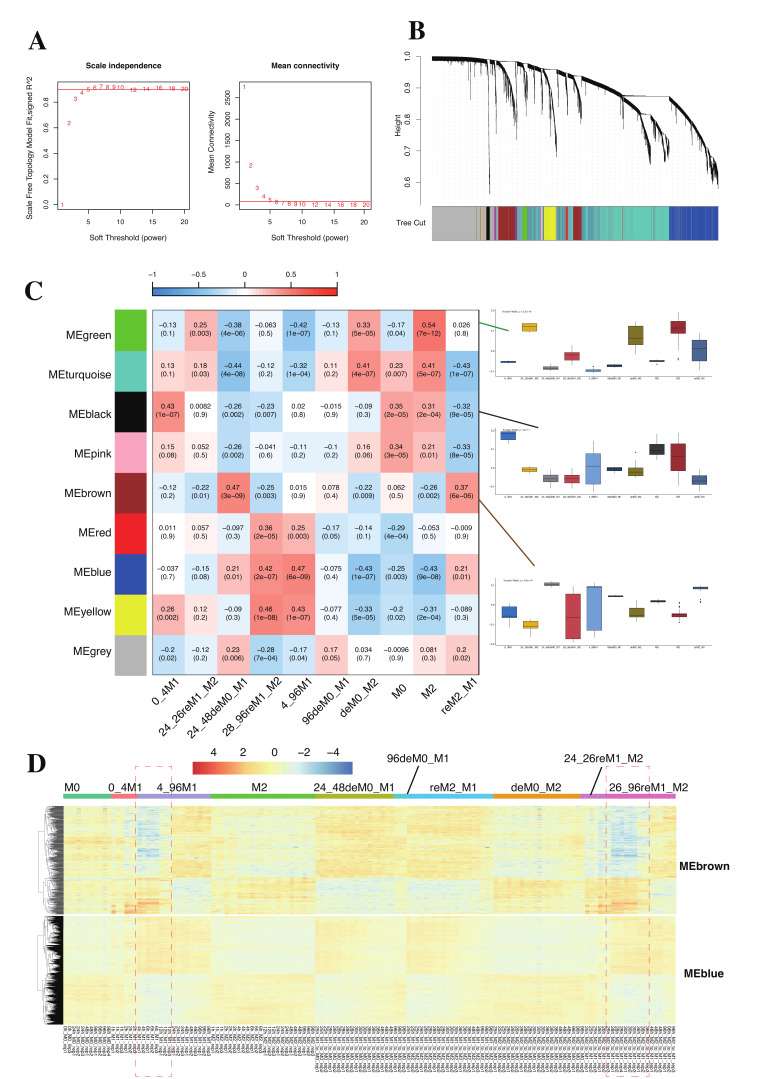
Co-expression network analysis based on WGCNA. (**A**) Analysis of scale-free fit index and mean connectivity for best parameter screening, the numbers represent different parameters. (**B**) Cluster dendrogram of co-expression genes with co-expression modules, the colors represent different gene modules. (**C**) Heatmap of associations among module eigengenes with all identified co-expression modules, the colors represent different gene modules. On the right side, bar plots are used to display the correlation coefficients between MEgreen, MEblack, and MEbrown with all phenotypes. (**D**) The heatmap shows the expression of MEbrown genes and MEblue genes across all samples. The portion highlighted by the red dashed circle indicates the occurrence of a late on-site phenomenon for brown genes in the 4_96M1 group and the 26_96reM1_M2 group.

Further, using the reclassified RNA expression data, we utilized WGCNA to analyze the relationship between the obtained gene expression matrix and the nine macrophage polarization phenotypes. We chose a soft thresholding power of 6, and the scale-free fit index (signed R^2^) was 0.9 (Figure 4A) and adjusted the minimum module size (minModuleSize) to 30 genes and then merged modules with a height cut-off (mergeCutHeight) less than 0.25. In this manner, we selected ten independent co-expression modules based on the generated hierarchical clustering dendrogram (Figure 4B), specifically named MEblack (51 genes), MEblue (1084 genes), MEbrown (466 genes), MEgreen (144 genes), MEpink (39 genes), MEred (69 genes), MEturquoise (1770 genes), and MEyellow (203 genes), as significant gene modules for downstream analysis. MEgrey with 1174 genes represents unmapped genes in this context (Figure 4C). Through an investigation of these modules’ eigengenes and eigengene associations, we found that despite their independence, MEgreen, MEturquoise, MEblack, and MEpink were relatively highly correlated. MEbrown, MEred, MEblue, and MEyellow were also relatively highly correlated (Figure S3). Subsequently, we computed the correlation of each independent module with each macrophage phenotype (Figure 4C). To assess the correlation between gene clusters and M1/M2 hysteresis, we focused on phenotypes with depolarization or repolarization history. For instance, we observed that MEgreen shows a relatively high correlation with the deM0_M2 and M2 phenotypes, while it is not correlated with the M0 phenotype. This suggests that the genes in MEgreen retain the expression pattern of the M2 phenotype within depolarized M0 cells, indicating the presence of M2 hysteresis. Thus, MEgreen is associated with M2 hysteresis in M0 cells. Using a similar approach, we found that the clusters MEgreen, MEturquoise, MEblack, and MEpink exhibited strong correlations with the M0, M2, deM0_M2, and incompletely polarized phenotypes (0_4M1 and 24_26reM1_M2). These genes represent the features associated with the M0-like and M2-like phenotypes. We observed that MEgreen and MEturquoise are associated with early M1 repolarized (24_26reM1_M2) and M0 depolarized M2 (deM0_M2) hysteresis. However, as the stimulation duration increases, the correlation with M2 hysteresis diminishes following prolonged M1 repolarization, as evidenced by the low correlation observed between these two gene clusters and the late M1 repolarized macrophages (28_96reM1_M2). This aligns with our previous research findings that the intensity of M1 hysteresis is significantly larger than that of M2 hysteresis, and it is challenging to clearly define M2 hysteresis genes and chromatin regions [9].

On the other hand, the clusters MEbrown, MEred, MEblue, and MEyellow showed relatively high correlations with M1-like phenotypes (28_96reM1_M2, 4_96M1) and phenotypes displaying M1 hysteresis (24_48deM0_M1, reM2_M1). We specifically focused on the clusters MEbrown and MEblue, noting that MEbrown exhibits a stronger correlation with M1 hysteresis phenotypes (24_48deM0_M1 and reM2_M1) compared to MEblue. However, MEbrown does not show a correlation with 4_96M1 and 28_96reM1_M2, which represent M1-like phenotypes. Upon comparing the gene expression profiles of these two gene clusters, we observed that, unlike the relatively consistent expression of genes in MEblue, most genes in MEbrown exhibit either high or low expression after more than 12 h of M1 polarization and repolarization (Figure 4D). The initiation of polarization significantly impacts the RNA expression data from 0 to 12 h, thereby affecting the results of the correlation analysis. This confirms that MEbrown also has a strong correlation with M1 cells and repolarized M1 cells stimulated for more than 12 h. It again underscores the necessity of reclassifying macrophage phenotypes based on stimulation duration.

In summary, we identified two gene clusters, MEbrown and MEblue, that are significantly associated with M1 hysteresis. Notably, genes within the MEbrown cluster exhibit a late-onset phenomenon, distinguishing them from those in the MEblue cluster.

### 2.4. GO Term and KEGG Enrichment Analysis Unveiled the Functions of Identified Gene Modules

**Figure 5:**
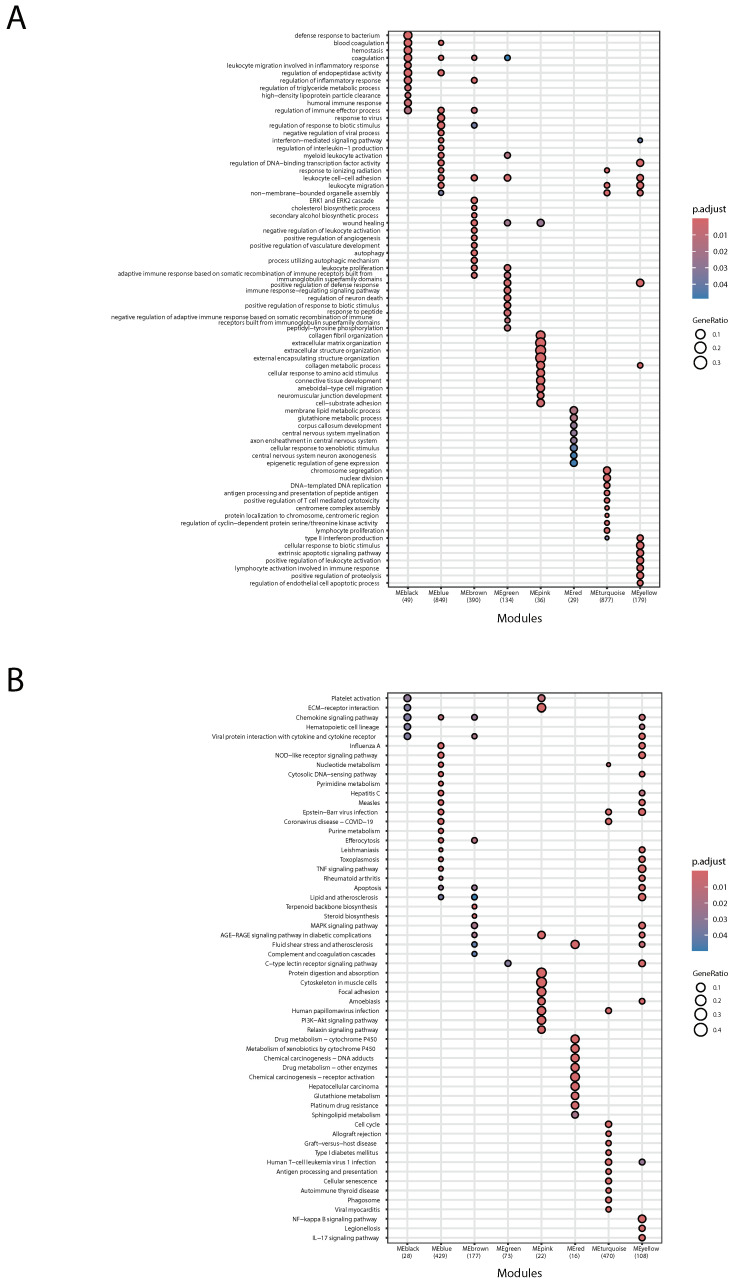
(**A**) GO and (**B**) KEGG analysis of all identified gene modules.

To further explore the functions of the identified gene modules by WGCNA, we performed GO term analysis (Figure 5A) and KEGG enrichment analysis (Figure 5B). The results revealed that all eight modules exhibited enrichments in specific GO terms and KEGG pathways. We observed that in the gene modules associated with M0 and M2, the top enriched biological processes and pathways for MEblack predominantly involve immune-related functions. These include defense response to the bacterium, regulation of inflammatory response, regulation of immune effector process, the chemokine signaling pathway, and viral protein interaction with cytokine and cytokine receptor. MEpink is primarily associated with functions related to the extracellular matrix, including extracellular matrix and structure organization, connective tissue development, cell–substrate adhesion, and focal adhesion. It is noteworthy that MEgreen and MEturquoise, which are associated with M2 hysteresis, are linked to leukocyte proliferation, chromosome segregation, nuclear division, DNA replication, and the cell cycle. This is consistent with our previous research findings, which concluded that M2 hysteresis potentially affects the cell cycle [9]. Subsequently, we examined gene modules highly associated with the M1-like phenotype. MEyellow represents hallmark M1 immunity and inflammatory responses, including, but not limited to, the NOD-like receptor signaling pathway, TNF signaling pathway, MAPK signaling pathway, NF-κB signaling pathway, and IL17 signaling pathway. MEred is associated with metabolic functions. Within the modules related to M1 hysteresis, MEblue is associated with M1 immune functions, including the regulation of immune effector processes, response to viruses, and interferon-mediated signaling pathways.

Since the results of the functional analysis indicated that MEblue is significantly associated with interferon-mediated signaling pathways (with an adjusted *p*-value = 2.42 × 10^−17^), we investigated the expression trends of all hysteresis genes within MEblue. We found that 13 ISGs (including Ccl5, Isg15, Ifi44, Ifi47, Ifi205, Ifit1, Ifit2, Ifit3, Ifitm1, Oasl1, Oasl2, Oas3, and Mx1) exhibited hysteresis phenomena [9] in MEblue (Figure S4). Additionally, significant hysteresis genes including Ccr3 and Lcn2 were also identified within the MEblue module, indicating a strong correlation between MEblue and M1 hysteresis. MEbrown, which is another gene cluster that is significantly associated with M1 hysteresis, is enriched in pathways such as cholesterol and secondary alcohol biosynthesis, lipid metabolism, and atherosclerosis, as well as steroid biosynthesis. Additionally, four M1 hysteresis genes (Fn1, Timp1, Dcstamp, Serpinb2) were identified in MEbrown (Figure S5). These genes exhibit sustained non-expression or low expression following M1 stimulation. The above finding preliminarily confirms our hypothesis that M1 hysteresis in macrophages influences their lipid metabolism.

### 2.5. Lipid Biosynthesis-Related Genes Drive the Expression of MEbrowm to Influence Macrophage Function in Lipid Metabolism

**Figure 6:**
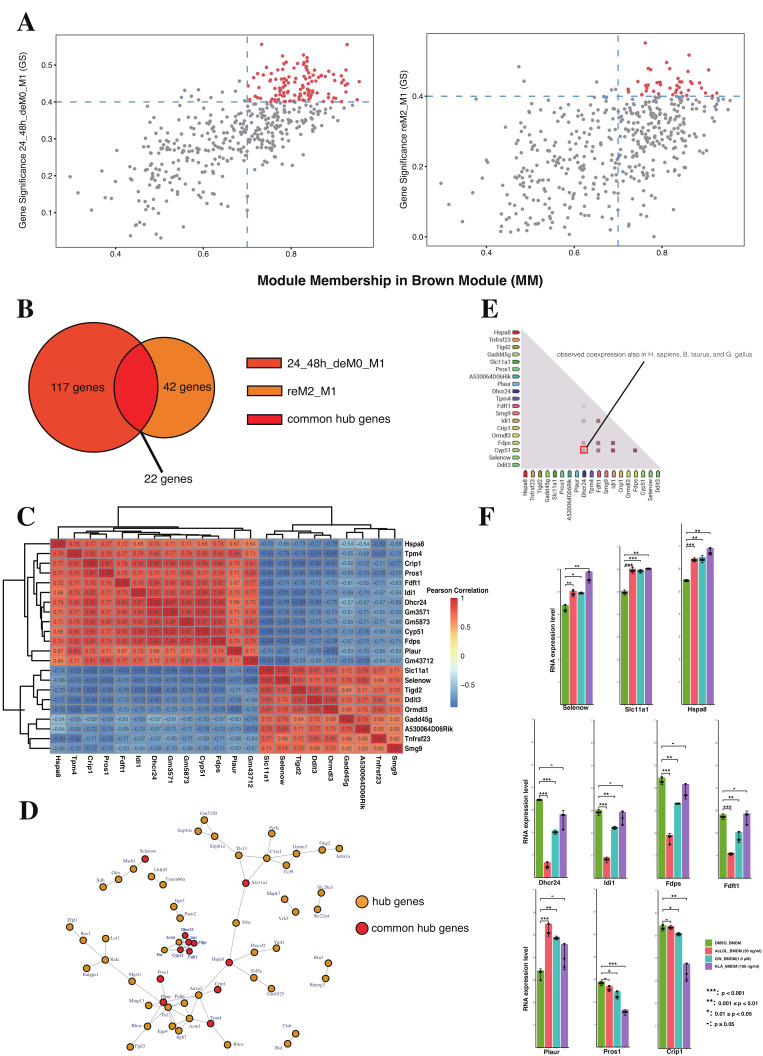
Identifying core sub-network and hub genes for module MEbrown. (**A**) Scatter plot of module membership and gene significance in the brown module to 24_48h_deM0_M1 and reM2_M1 phenotypes, the dots represent all genes in gene module. (**B**) Venn diagram representing number of hub genes of MEbrown to 24_48h_deM0_M1 and reM2_M1 and their common hub genes. (**C**) Pairwise correlation analysis of 22 common hub genes shows a significant positive or negative correlation between each hub gene in heatmap. (**D**) Protein–protein interaction (PPI) network of hub genes was identified. Yellow nodes represent uncommon hub genes and red nodes represent common hub genes. (**E**) Experimental based pairwise gene co-expression correlation identified in the STRING database. (**F**) Bar plots illustrating differences in RNA expression (TPM) of hub genes after treated by AcLDL, GW3965, and KLA. The asterisks indicate that the differences are statistically significant. ‘*’ *p* < 0.05, ‘**’ *p* < 0.01, ‘***’ *p* < 0.001. ‘-’ not significant.

The notable differences in gene expression confirm our findings in the functional analysis section, indicating a close association between MEblue and M1 immune functions and interferon-mediated signaling pathways. Lisa Willemsen et al. [19] previously reported significant expression changes in these ISGs in lipid-loaded samples. The expression of ISGs in macrophages may be related to intracellular lipid levels. Based on our functional analysis, we speculate that the regulatory factors present in the MEbrown cluster may influence macrophage lipid metabolism and, consequently, affect lipid levels within macrophages. To address this issue, we independently screened MEbrown genes for hub genes, which are highly connected to other genes in the gene regulatory network and are crucial for the overall regulation of the network, specifically in association with the 24_48h_deM0_M1 and reM2_M1 phenotypes. We utilized MM (module membership) > 0.7 and GS (gene significance) > 0.4 as filtering criteria for hub genes (Figure 6A). We found 117 genes and 42 genes as hub genes for the 24_48h_deM0_M1 and reM2_M1 phenotypes, respectively, within MEbrown. Through comparison, we identified 22 common hub genes between the described two phenotypes, including Hspa8, Tpm4, Crip1, Pros1, Fdft1, Idi1, Dhcr24, Gm3571, Gm5873, Cyp51, Fdps, Plaur, Gm43712, Slc11a1, Selenow, Tigd2, Ddit3, Ormdl3, Gadd45g, A530064D06Rik, Tnfrsf23, and Smg9 (Figure 6B). After calculating the Pearson correlation coefficients between the expression levels of these genes across all 144 samples, we found that these genes exhibit significantly high correlations with each other, distinctly forming two groups with either positive or negative correlations (Figure 6C). Then, we investigated the interactions of these genes in the PPI network (Figure 6D). Notably, a sub-network including the proteins Dhcr24, Idi1, Fdps, Fdft1, Cyp51, Sc5d, and Por was identified, with four of these proteins being common M1 hysteresis hub genes that we screened for. Among them, Dhcr24 (24-dehydrocholesterol reductase), squalene synthase Fdft1 (farnesyl diphosphate farnesyltransferase 1), and Cyp51 (cholesterol biosynthetic enzyme lanosterol 14α-demethylase) have all been reported to have significant impacts on cholesterol biosynthesis [22]. Similarly, Idi1 has been confirmed to play a key role in the cholesterol biosynthesis pathway and lipid metabolism synthesis [23]. Fdps is another key cholesterol biosynthetic gene that has always been induced in colon cancer tissues [24]. Also, the inhibition of POR activity would halt the production of cholesterol and its breakdown into bile acids [25]. Sc5D (sterol-C5-desaturase), which encodes an enzyme involved in cholesterol biosynthesis, converts lathosterol to 7-dehydrocholesterol in the cholesterol biosynthesis pathway [26]. The evidence suggest that this sub-network of hub genes plays a significant role in lipid metabolism and cholesterol biosynthesis.

Subsequently, we screened the co-expression data from the STRING database and found that, among all common hub genes, only the co-expression relationships involving Fdft1, Idi1, Fdps, Cyp51, and Dhcr24 have been consistently observed across numerous experiments in the database. Specifically, the co-expression of Cyp51 and Dhcr24 has been observed not only in Mus musculus but also in Homo sapiens, Bos taurus, and Gallus gallus based on the record from the string database, highlighting the conservation co-expression relationship between Cyp51 and Dhcr24 across different species (Figure 6E). The roles of these hub genes in lipid metabolism were further confirmed in lipid-loaded macrophages which we collected from the GEO database. By comparing with the RNA expression level of DMSO-treated BMDM, significant expression differences were observed during lipid loading processes (AcLDL, GW3965, and KLA) for Selenow, Slc11a1, Hspa8, Dhcr24, Idi1, Fdps, Fdft1, Plaur, Pros1, and Crip1 (Figure 6F). The expression of genes related to cholesterol biosynthesis, including Dhcr24, Idi1, Fdps, and Fdft1, was suppressed in lipid-loaded samples. This suggests that the accumulation of externally ingested lipids in macrophages significantly suppresses the expression of cholesterol biosynthesis-related genes, reducing endogenous lipid synthesis to maintain cellular lipid levels [27].

In summary, our results further confirm that the hub genes of MEbrown are significantly associated with macrophage lipid metabolism and cholesterol biosynthesis. These genes drive the overall gene expression of the MEbrown module to influence macrophage function in lipid metabolism.

### 2.6. Hysteresis of Marco (SR-A6) and Abca1/Abca2 Results in Elevated Intracellular Lipid Levels and Suppression of Lipid Biosynthesis-Related Genes

**Figure 7:**
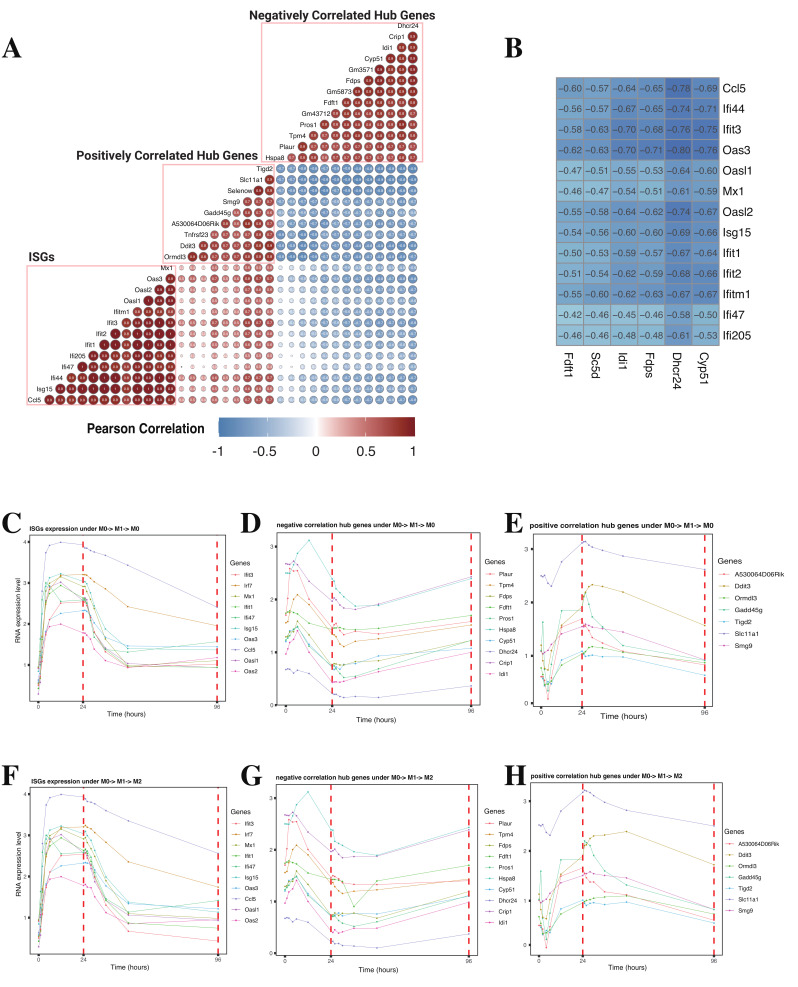
Strong gene expression correlation between ISGs and hub genes. (**A**) Correlation coefficients between ISGs and hub genes selected from MEbrowm. The values and transparency represent Pearson’s correlation coefficients of each gene pair. (**B**) Correlation coefficients between ISGs and lipid biosynthesis-related genes. Heatmap of top 30 genes with the highest connectivity identified in MEmagenta and MEred across all samples. (**C**–**H**) Gene expression levels of the ISGs, negative correlation hub genes, and positive correlation hub genes under (**C**–**E**) M0 → M1 → M0 and (**F**–**H**) M0 → M1 → M2 from 0 h to 96 h.

Due to the abnormal expression of ISGs observed in lipid-loaded macrophages in Lisa Willemsen et al.’s study [19], and our previous finding of ISG hysteresis in macrophages with a history of M1 polarization [9], we hypothesize that ISGs may be related to the expression of lipid biosynthesis-related genes, which are also the hub genes regulating the MEbrown module that we identified in M1 hysteresis. To investigate their relationship, we first calculated the Pearson correlation coefficients between the RNA expression levels of ISGs and hub genes across all macrophage polarization datasets. We found that, regardless of the stimulus condition, subsets of hub genes were significantly positively or negatively correlated with the expression of ISGs (Figure 7A). Interestingly, we found that the hub genes associated with cholesterol biosynthesis, including Cyp51, Dhcr24, Fdps, Idi1, Sc5d, and Fdft1, exhibited a significantly negative correlation with the expression levels of all ISGs (Figure 7B). To investigate the negatively correlated gene expression during the repolarization process, we examined the expression profile of ISGs (Figure 7C,F), negatively correlated hub genes (Figure 7D,J), and positively correlated hub genes (Figure 7E,H) in the transitions of M0 → M1 → M0 and M0 → M1 → M2 in 96 h. We found that, unlike the positively correlated hub genes which maintain a relatively consistent expression trend with ISGs, the negatively correlated hub genes exhibited a significant decrease in expression 6 to 12 h after M1 stimulation. During the de-/repolarization process, these genes displayed hysteresis, remaining at relatively low expression levels and not fully recovering even after 96 h. This trend is opposite to the ISGs, which exhibit sustained high expression that gradually decreases during de-/repolarization.

**Figure 8:**
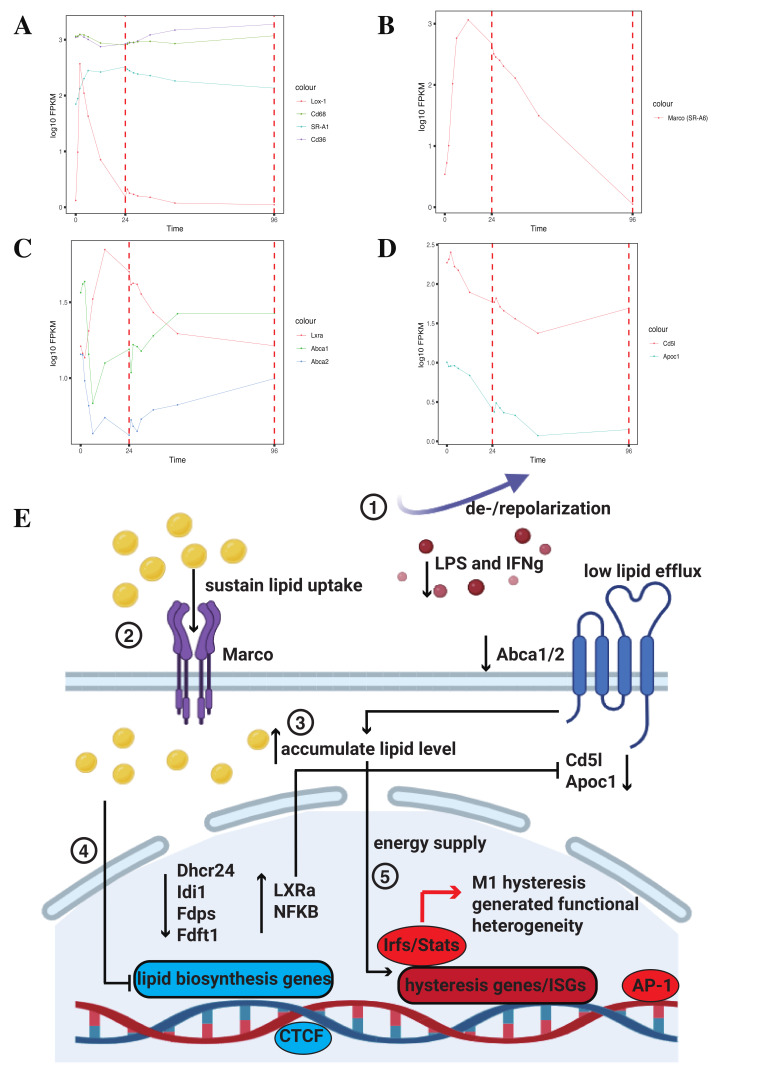
Strong gene expression correlation between ISGs and hub genes. (**A**–**D**) Gene expression levels of (**A**) Lox-1, Cd68, SR-A1, and Cd36; (**B**) Marco (SR-A6); (**C**) Lxra, Abca1, and Abca2; and (**D**) Cd5l and Apoc1 under M0 → M1 → M0 between 0 and 96 h. (**E**) Schematic representation of the relationship between macrophage M1 hysteresis and macrophage lipid metabolism with the order of protentional underlying mechanism (1–5).

From the above results, we found that during the occurrence of M1 hysteresis in macrophages, the expression of cholesterol biosynthesis-related genes remains suppressed. In contrast, pro-inflammatory genes, represented by ISGs, continue to be expressed. This suggests that the main energy source driving the pro-inflammatory response associated with macrophage M1 hysteresis is not endogenous cholesterol biosynthesis. Studies indicate that another major pathway for increasing lipid levels in macrophages is through the direct uptake of lipids from the extracellular environment [27]. To further investigate how cells replenish lipid levels during the M1 hysteresis process to secure energy sources, we examined the expression of scavenger receptors which facilitate macrophage lipid uptake to a large extent [20]. It has been reported that besides serving as pattern recognition receptors and being known to act in coordination with other co-receptors such as Toll-like receptors to generate immune responses, a critical function of scavenger receptors is to uptake LDL, VLDL, and oxidized lipoproteins [28]. After being digested in the lysosome, this process increases the levels of free cholesterol and free fatty acids in the cell, subsequently promoting the activation of multiple downstream transcription factors (TFs) including LXRs, PPARs, SREBPs, and NF-κB to enhance inflammatory responses [27]. We examined the expression profiles of several major scavenger receptors, including Class A receptors SR-A1 and Marco (SR-A6), Class B receptor Cd36, Class D receptor Cd68, and Class E receptor Lox-1. We observed that SR-A1, Cd36, and Cd68 maintained high expression patterns similar to the unpolarized state following M1 stimulation, while Lox-1 quickly returned to its original low expression pattern after M1 stimulation under M0 → M1 → M0 (Figure 8A) and M0 → M1 → M2 (Figure S6). Interestingly, Marco exhibited a hysteresis effect during the depolarization process, only returning to its original expression level after 96 h under M0 → M1 → M0 (Figure 8B) and M0 → M1 → M2 (Figure S7). Previous research has shown that in both human and conditional mouse models of prostate cancer with macrophage infiltration, the overexpression of Marco is linked to lipid intake and lipid droplet accumulation, while the inhibition of Marco promotes tumor suppression in these models [29]. So, the hysteresis in Marco gene expression may lead to an increase in macrophage lipid intake and accumulation of intracellular lipid levels.

Previous studies have shown that lipid homeostasis in macrophages is regulated at multiple levels, including receptor-mediated uptake, metabolism, and efflux [30]. To determine how cholesterol efflux-related genes are expressed during M1 hysteresis and how they influence intracellular lipid levels, we examined the expression of Lxra and its target genes Abca1/Abca2, which are critical factors that regulate the efflux of free cholesterol [27]. A lack of functional Abca2 generates abnormalities in intracellular lipid distribution/trafficking in macrophages [31]. We found that Lxra remained continuously activated after M1 stimulation, even maintaining a period of hysteresis during depolarization under M0 → M1 → M0 (Figure 8C) and M0 → M1 → M2 (Figure S8). And Lxra’s target genes, Abca1 and Abca2, exhibited sustained repression post-stimulation and did not fully return to their initial expression states even after 96 h under M0 → M1 → M0 (Figure 8D) and M0 → M1 → M2 (Figure S9). The decrease in the expression of Abca1 and Abca2 would reduce lipid efflux in macrophages, maintaining high intracellular lipid levels. To further validate the decrease in lipid efflux in macrophages, we examined two other important Lxr targets, Cd5l and Apoc1, which are highly associated with macrophage lipid transportation [32,33,34]. We observed that these two genes consistently exhibited reduced expression following M1 stimulation and maintained a state of hysteresis (Figure 8D).

Thus, we determined that in M1 hysteresis, the expression of cholesterol biosynthesis-related genes is suppressed, while the upregulation of Marco leads to increased lipid uptake. Additionally, the low expression of genes such as Abca1/2, Cd5l, and Apoc1 results in inhibited lipid efflux, providing a sufficient energy supply for the activation of IFN signaling driven by the high expression of ISGs and other pro-inflammatory genes we found in M1 hysteresis.

## 3. Discussion

Extensive prior research, as well as our studies, have confirmed that macrophages, like adaptive immune cells, can develop an immunological memory, which is reflected not only in gene expression but also in epigenetic modifications. In previous studies, we utilized in vitro data from BMDM polarization and depolarization to ascertain significant M1 hysteresis in vitro, a phenomenon closely associated with the regulation of different chromatin regions by the AP-1 family and CTCF. We are aware that sustained M1 inflammatory responses require external lipid intake or endogenous biosynthesis to continue as a source of energy. Concurrently, we observed a hysteresis phenomenon in the gene expression of numerous ISGs, which may be linked to lipid metabolism. We propose that macrophage hysteresis may be associated to some extent with macrophage lipid metabolism.

In this study, we reanalyzed RNA-seq data from macrophages during polarization and depolarization over a period of 0 to 96 h, incorporating RNA-seq from lipid-loaded macrophages for comparative validation. Our findings include the following: 1. The macrophages used in our study demonstrated substantial plasticity and extreme sensitivity to the duration of stimulation, emphasizing their dynamic nature. 2. Using WGCNA, we identified two gene clusters, MEblue and MEbrown, that are significantly associated with M1 hysteresis. While MEblue genes are enriched in M1 immune functions and interferon-mediated signaling pathways, MEbrown genes exhibited a delayed onset of upregulation or downregulation, occurring after 12 h of stimulation, and are highly associated with lipid metabolism, cholesterol biosynthesis, and secondary alcohol biosynthesis. 3. Further analysis of the MEbrown module revealed 22 core driver hub genes, including Dhcr24, Idi1, Fdps, Fdft1, and Cyp51, which play crucial roles in cholesterol biosynthesis. These genes showed either significant positive or negative correlations within the co-expression network and demonstrated robust protein–protein interaction (PPI) relationships. Notably, these genes were significantly downregulated in lipid-loaded samples. 4. A deeper investigation into the relationship between cholesterol biosynthesis-regulating hub genes and interferon-stimulated genes (ISGs) uncovered significant negative correlations, highlighting potential regulatory interactions. 5. During the M1 hysteresis process, the scavenger receptor Marco, which mediates macrophage lipid uptake, exhibited a hysteretic RNA expression pattern. Additionally, lipid efflux regulators such as Abca1 and Abca2, along with their upstream mediators, displayed persistently low expression levels. This coordinated expression pattern compensates for the reduced intracellular cholesterol biosynthesis, ensuring lipid homeostasis and providing the energy necessary to sustain pathways such as interferon (IFN) signaling.

Integrating these findings, we can conclude our hypothesis that following depolarization or repolarization, the sustained high expression, i.e., hysteresis, of the scavenger receptor Marco on the macrophage surface membrane leads to higher intracellular lipid uptake. Concurrently, the continuous expression of the upstream mediator Lxra leads to the suppression of genes such as Abca1, Abca2, Cd5l, and Apoc1, which are in charge of lower lipid efflux in macrophages. Studies have shown that lipids uptaken by macrophages are broken down into free cholesterol (FC) and fatty acids (FAs), which are then transported to the endoplasmic reticulum (ER) membrane for further processing. When deregulated lipid uptake occurs, the excessive accumulation of FC and other lipids in the ER activates sensors of the unfolded protein response, triggering ER stress, which in turn regulates the gene expression on the lipid uptake, efflux, and biosynthesis [30]. So, it is highly likely that the increase in lipid uptake, combined with the reduction in lipid efflux, leads to lipid accumulation within the cells. Elevated lipid levels subsequently influence gene expression, resulting in the suppression of genes involved in lipid biosynthesis, such as Dhcr24, Idi1, Fdps, and Fdft1, to balance overall intracellular lipid levels. This phenomenon was also confirmed in our validation analysis using data from lipid-loaded samples. The accumulated intracellular lipids then serve as an energy source for the inflammatory responses generated by M1 hysteresis. Ultimately, this results in the activation of regulators such as IRFs, STATs, AP-1, and CTCF in M1 hysteresis, contributing to cellular functional heterogeneity, which has been extensively explored in our previous research [9] (Figure 8E).

In recent years, the role of lipid metabolism has been extensively studied within the classic M1/M2 macrophage polarization paradigm. Numerous studies underscore the critical role of lipid metabolism in inflammatory macrophage polarization, supporting proper inflammatory responses through membrane remodeling and as precursors for various inflammatory mediators [33,34,35,36]. Lipids, sourced from diverse origins such as native LDL, oxidized LDL particles, or free fatty acids, and existing in different forms, are internalized by macrophages through various scavenger receptors, including CD36, MARCO, and SR-A1. Once inside the cell, most lipids—including those bound to lipoproteins and fatty acids—are transported to the lysosomal–endosomal compartment. Here, lysosomal acid lipase (LAL) catalyzes the conversion of LDL-derived cholesteryl esters into fatty acids and free cholesterol, which are then transferred to the endoplasmic reticulum where excess lipids are stored in lipid droplets [37,38,39]. These lipids continuously provide energy to the macrophage. Additionally, macrophages can actively export lipids through ATP-binding cassette (ABC) transporters. Clinically, an increase in the expression of the cholesterol transporter ABCA1 in macrophages, and in the liver, which enhances cholesterol efflux, is always indicative of an anti-atherosclerotic effect [30,31]. Both lipid scavenging receptors and ABC transporters play pivotal roles in dictating the metabolic fates of scavenged lipids [40,41,42,43,44]. Zhu et al. demonstrated in a macrophage-specific Abca1 knock-out mouse model that both in vitro and in vivo macrophages exhibited a significant increase in free cholesterol (FC) and membrane lipid rafts. Upon LPS stimulation, these macrophages produced elevated levels of pro-inflammatory cytokines and showed enhanced activation of the NF-κB and MAPK pathways [45]. This work supports our observations regarding macrophage hysteresis and its connection to lipid metabolism. The sustained interferon response and enrichment of pathways such as NF-κB observed in depolarized/repolarized M1 macrophages following LPS stimulation [9] are likely driven by lipid accumulation caused by factors like ABCA1.

Multiple lines of evidence substantiate the concept that lipid remodeling following macrophage activation is crucial for maintaining appropriate inflammatory responses and host defense [46,47,48]. Certain lipids also function as precursors for the production of inflammatory lipid mediators [49]. MARCO, a Class A scavenger receptor, is widely expressed across various myeloid populations, including peritoneal, alveolar, splenic macrophages, Kupffer cells, and dendritic cells [50,51,52]. It is highly induced by stimuli such as LPS or granulocyte/macrophage colony-stimulating factor (GM-CSF) [53], which are associated with lipid accumulation during M1 hysteresis. The rapid induction of MARCO expression in response to infectious stimuli plays a pivotal role in mediating appropriate Toll-like receptor (TLR)-dependent inflammatory responses [52,54]. Further, evidence indicates that lipid-loaded macrophages exhibit heightened MARCO surface expression. Notably, murine and human tumor-associated macrophages (TAMs) show elevated MARCO expression, potentially linked causally to their increased lipid accumulation [55,56,57]. Experiments demonstrate that the accumulation of modified and oxidized lipids is a primary driver of inflammatory responses and is implicated in numerous diseases, including atherosclerosis, steatohepatitis, obesity-induced insulin resistance, and neurodegeneration [57,58,59]. In several cancer model experiments, the high expression of MARCO has been shown to promote lipid accumulation confers protumor features to tumor-associated macrophages. Targeting lipid accumulation through MARCO blockade not only hinders tumor growth and invasiveness but also enhances the efficacy of chemotherapy in these cancer models [29,60,61]. While these studies collectively validate our findings, several aspects still require attention, such as potential batch effects due to variations in experimental conditions across different datasets, the limitation of RNA-seq data capturing only the endpoints of de-/repolarization at 96 h, and as all the data were derived from mouse BMDM in vitro conditions, the results may not directly correspond to tissue-resident macrophages in vivo conditions. Therefore, further data and experimental validation are needed in the future to deepen our understanding of the hysteresis phenomenon in macrophages.

## 4. Materials and Methods

### 4.1. Data Preparation

We collected a total of 162 sets of bulk RNA-seq data from polarized/depolarized/repolarized BMDM (bone marrow-derived macrophage) (GSE158094) and lipid-loaded macrophage (GSE118656, GSE193118) from the NCBI Gene Expression Omnibus (GEO). For the polarized/depolarized/repolarized data, BMDMs were treated and polarized/depolarized/repolarized with either M1 stimulus (LPS + IFNγ) or M2 stimulus (IL-4). For the lipid-loaded data, BMDMs were treated by DMSO (dimethyl sulfoxide, a chemical that dissolves organic and inorganic substances), 1.0 μM GW3965 (a selective liver X receptor agonist), 100 ng/mL KLA (a natural endotoxin and a combination of oligosaccharide and lipid), and 50 μg/mL AcLDL (acetylated low-density lipoprotein). We categorized the RNA-seq data from macrophage polarization and lipid-loaded macrophages according to different stimulation conditions and obtained eight distinct groups: lipid (all lipid-loaded macrophages described in the method section), M0 (unpolarized macrophages), M1 (M1 macrophages polarized from M0), M2 (M2 macrophages polarized from M0), deM0_M1 (depolarized M0 from M1), deM0_M2 (depolarized M0 from M2), reM2_M1 (repolarized M2 from M1), and reM1_M2 (repolarized M1 from M2) for downstream analysis.

### 4.2. Sequencing Data Preprocessing

We assessed the quality of all the sequence data using FastQC (v0.12.1) [62] and MultiQC (v1.26) [63]. After confirming the quality of the sequence data, we employed Trim Galore (v0.6.10) [64] to remove low-quality reads and process adaptors. Following a thorough quality check, we used STAR (v2.7.11) [65] to map the processed FASTQ files to the mm10 reference genome. Subsequently, we utilized the cuffnorm functionality within Cufflinks (v2.2.1) [66] to obtain normalized FPKM values for genes.

### 4.3. Batch Effect Correction, Quality Control, and Data Processing

We initially transformed the gene’s FPKM (Fragments Per Kilobase of transcript per Million mapped reads) expression matrix into a TPM (transcripts per million) matrix. Using the 3167 mouse housekeeping genes (https://housekeeping.unicamp.br/Housekeeping_Genes_Mouse.RData, accessed on 4 October 2024) as a background, we applied RUVSeq (v3.20) [67] to remove batch effects from bulk RNA-seq data originating from different projects. Through further analysis, including the measurement of relative gene expression levels across all samples and examination of the distribution of PCA, we confirmed the successful elimination of batch effects. Subsequently, we calculated and kept the top 5000 genes with the highest Median Absolute Deviation (MAD) for downstream analysis.

### 4.4. Weighted Gene Co-Expression Network Analysis (WGCNA)

Based on the processed gene expression matrix, we constructed co-expression networks using WGCNA (v1.73) [68] and performed gene co-expression analysis to determine the correlation between gene modules and macrophage phenotypes. After conducting the hierarchical clustering of samples to remove outliers, in order to ascertain the link strengths between two genes, a co-expression network was first built using a suitable soft thresholding power (6, scale-free R^2^ = 0.9), chosen in accordance with scale-free fitting. Then, the link between gene pair expression, or the Topological Overlap Matrix (TOM), is defined to reflect the similarity between genes at both the expression and network topology levels. The dissimilarity matrix (1-TOM) derived from the TOM was constructed as an additional adjacency matrix that takes topological similarity into account. By using a sensitive module detection parameter of 3 and a minimal module size of 30, hierarchical clustering of genes was used to identify important modules. High similarity gene modules were found and combined (height threshold of 0.25). Potential hub genes correlated with M1 hysteresis clusters (GS > 0.4 and MM > 0.7) were identified for downstream analysis using gene significance (GS), which is defined as the association with each gene expression level and the clusters, and the module membership (MM), which is defined as the association with the gene expression profile and the module eigengene.

### 4.5. Gene Ontology Analysis and Pathway Analysis

To elucidate the functions of different gene modules, we conducted enrichment analysis for Gene Ontology (GO) terms and the Kyoto Encyclopedia of Genes and Genomes (KEGG) pathways using clusterProfiler (v4.14.4) [69].

### 4.6. Other Bioinformatics Analysis and Statistical Analysis

We used the protein–protein interaction (PPI) and gene co-expression data from the STRING database (v12.0) [70]. We calculated the Pearson correlation coefficient with hub gene expression and generated the PPI network graph using the igraph package (v2.1.2.9003) [71]. Benjamani–Hochberg correction was used to compute the *p*-values. Statistical significance was defined as *p* < 0.05. The remaining *p*-value results were as follows: “-”: *p* ≥ 0.05, “*”: *p*< 0.05, “**”: *p*< 0.01, and “***”: *p*< 0.001.

## 5. Conclusions

In conclusion, our findings demonstrate that macrophage depolarization or repolarization induces sustained high expression (hysteresis) of the scavenger receptor Marco, which enhances intracellular lipid uptake. Simultaneously, persistent expression of the upstream mediator Lxra suppresses genes associated with lipid efflux (e.g., Abca1, Abca2, Cd5l, and Apoc1), leading to lipid accumulation. This excessive lipid uptake likely triggers endoplasmic reticulum (ER) stress through the activation of the unfolded protein response, further altering lipid-related gene expression to restore intracellular lipid balance. The accumulated lipids fuel inflammatory responses serve as an energy source for the inflammatory responses generated by M1 hysteresis, activating key regulators such as IRFs, STATs, AP-1, and CTCF, which contribute to the functional heterogeneity of macrophages. These findings highlight a potential regulatory interplay between M1 macrophage hysteresis and lipid metabolism, offering insights into the mechanisms underlying innate immune memory. Immune memory generated via hysteresis or trained immunity plays a pivotal role in combating infectious diseases. Further exploration of the connection between macrophage hysteresis and lipid metabolism enriches our understanding of this unique form of immune memory. However, our findings warrant additional validation in in vivo settings or tissue-resident macrophages. Future research should aim to unravel the detailed mechanisms of hysteresis and its implications, potentially paving the way for the development of universal vaccines. Such vaccines could provide broad protection against diverse pathogens and be invaluable during future pandemics.

## Data Availability

Publicly available datasets were analyzed in this study. These data can be found here: GSE158094, GSE118656, and GSE193118.

## Supplementary Materials

The following supporting information can be downloaded at: https://www.mdpi.com/article/10.3390/ijms26010111/s1.

## References

[1] Davies L.C., Rosas M., Jenkins S.J., Liao C.-T., Scurr M.J., Brombacher F., Fraser D.J., Allen J.E., Jones S.A., Taylor P.R. (2013). Distinct Bone Marrow-Derived and Tissue-Resident Macrophage Lineages Proliferate at Key Stages during Inflammation. Nat. Commun..

[2] Martinez F.O., Gordon S. (2014). The M1 and M2 Paradigm of Macrophage Activation: Time for Reassessment. F1000Prime Rep..

[3] Sica A., Mantovani A. (2012). Macrophage Plasticity and Polarization: In Vivo Veritas. J. Clin. Invest..

[4] Ivashkiv L.B. (2013). Epigenetic Regulation of Macrophage Polarization and Function. Trends. Immunol..

[5] Ando M., Ito M., Srirat T., Kondo T., Yoshimura A. (2020). Memory T Cell, Exhaustion, and Tumor Immunity. Immunol. Med..

[6] Liu Q., Sun Z., Chen L. (2020). Memory T Cells: Strategies for Optimizing Tumor Immunotherapy. Protein Cell.

[7] Seifert M., Küppers R. (2016). Human Memory B Cells. Leukemia.

[8] Bekkering S., Domínguez-Andrés J., Joosten L.A.B., Riksen N.P., Netea M.G. (2021). Trained Immunity: Reprogramming Innate Immunity in Health and Disease. Annu. Rev. Immunol..

[9] Zhang Y., Yang W., Kumagai Y., Loza M., Zhang W., Park S.-J., Nakai K. (2023). Multi-Omics Computational Analysis Unveils the Involvement of AP-1 and CTCF in Hysteresis of Chromatin States during Macrophage Polarization. Front. Immunol..

[10] Jeljeli M., Riccio L.G.C., Doridot L., Chêne C., Nicco C., Chouzenoux S., Deletang Q., Allanore Y., Kavian N., Batteux F. (2019). Trained Immunity Modulates Inflammation-Induced Fibrosis. Nat. Commun..

[11] Van Belleghem J.D., Bollyky P.L. (2018). Macrophages and Innate Immune Memory against Staphylococcus Skin Infections. Proc. Natl. Acad. Sci. USA.

[12] Weavers H., Evans I.R., Martin P., Wood W. (2016). Corpse Engulfment Generates a Molecular Memory That Primes the Macrophage Inflammatory Response. Cell.

[13] Xing Z., Afkhami S., Bavananthasivam J., Fritz D.K., D’Agostino M.R., Vaseghi-Shanjani M., Yao Y., Jeyanathan M. (2020). Innate Immune Memory of Tissue-Resident Macrophages and Trained Innate Immunity: Re-Vamping Vaccine Concept and Strategies. J. Leukoc. Biol..

[14] Netea M.G., Joosten L.A.B. (2018). Trained Immunity and Local Innate Immune Memory in the Lung. Cell.

[15] Vuscan P., Kischkel B., Joosten L.A.B., Netea M.G. (2024). Trained Immunity: General and Emerging Concepts. Immunol. Rev..

[16] Hardy T.M., Tollefsbol T.O. (2011). Epigenetic Diet: Impact on the Epigenome and Cancer. Epigenomics.

[17] Donohoe D.R., Bultman S.J. (2012). Metaboloepigenetics: Interrelationships between Energy Metabolism and Epigenetic Control of Gene Expression. J. Cell. Physiol..

[18] Hata M., Andriessen E.M.M.A., Hata M., Diaz-Marin R., Fournier F., Crespo-Garcia S., Blot G., Juneau R., Pilon F., Dejda A. (2023). Past History of Obesity Triggers Persistent Epigenetic Changes in Innate Immunity and Exacerbates Neuroinflammation. Science.

[19] Willemsen L., Chen H.-J., van Roomen C.P.A.A., Griffith G.R., Siebeler R., Neele A.E., Kroon J., Hoeksema M.A., de Winther M.P.J. (2022). Monocyte and Macrophage Lipid Accumulation Results in Down-Regulated Type-I Interferon Responses. Front. Cardiovasc. Med..

[20] Vogel A., Brunner J.S., Hajto A., Sharif O., Schabbauer G. (2022). Lipid Scavenging Macrophages and Inflammation. Biochim. Et Biophys. Acta (BBA) Mol. Cell Biol. Lipids.

[21] Liu S.X., Gustafson H.H., Jackson D.L., Pun S.H., Trapnell C. (2020). Trajectory Analysis Quantifies Transcriptional Plasticity during Macrophage Polarization. Sci. Rep..

[22] Liebergall S.R., Angdisen J., Chan S.H., Chang Y., Osborne T.F., Koeppel A.F., Turner S.D., Schulman I.G. (2020). Inflammation Triggers Liver X Receptor-Dependent Lipogenesis. Mol. Cell Biol..

[23] Chen X., Xiao C., Liu Y., Li Q., Cheng Y., Li S., Li W., Yuan J., Wang Y., Shen F. (2023). HUB Genes Transcriptionally Regulate Lipid Metabolism in Alveolar Type II Cells under LPS Stimulation. Heliyon.

[24] Gao S., Soares F., Wang S., Wong C.C., Chen H., Yang Z., Liu W., Go M.Y.Y., Ahmed M., Zeng Y. (2021). CRISPR Screens Identify Cholesterol Biosynthesis as a Therapeutic Target on Stemness and Drug Resistance of Colon Cancer. Oncogene.

[25] Porter T.D., Banerjee S., Stolarczyk E.I., Zou L. (2011). Suppression of Cytochrome P450 Reductase (POR) Expression in Hepatoma Cells Replicates the Hepatic Lipidosis Observed in Hepatic POR-Null Mice. Drug Metab. Dispos..

[26] Sugawara T., Fujimoto Y., Ishibashi T. (2001). Molecular Cloning and Structural Analysis of Human Sterol C5 Desaturase. Biochim. Biophys. Acta.

[27] Remmerie A., Scott C.L. (2018). Macrophages and Lipid Metabolism. Cell Immunol..

[28] Taban Q., Mumtaz P.T., Masoodi K.Z., Haq E., Ahmad S.M. (2022). Scavenger Receptors in Host Defense: From Functional Aspects to Mode of Action. Cell Commun. Signal..

[29] Masetti M., Carriero R., Portale F., Marelli G., Morina N., Pandini M., Iovino M., Partini B., Erreni M., Ponzetta A. (2021). Lipid-Loaded Tumor-Associated Macrophages Sustain Tumor Growth and Invasiveness in Prostate Cancer. J. Exp. Med..

[30] Sukhorukov V.N., Khotina V.A., Chegodaev Y.S., Ivanova E., Sobenin I.A., Orekhov A.N. (2020). Lipid Metabolism in Macrophages: Focus on Atherosclerosis. Biomedicines.

[31] Calpe-Berdiel L., Zhao Y., de Graauw M., Ye D., van Santbrink P.J., Mommaas A.M., Foks A., Bot M., Meurs I., Kuiper J. (2012). Macrophage ABCA2 Deletion Modulates Intracellular Cholesterol Deposition, Affects Macrophage Apoptosis, and Decreases Early Atherosclerosis in LDL Receptor Knockout Mice. Atherosclerosis.

[32] Fuior E.V., Gafencu A.V. (2019). Apolipoprotein C1: Its Pleiotropic Effects in Lipid Metabolism and Beyond. Int. J. Mol. Sci..

[33] Yan J., Horng T. (2020). Lipid Metabolism in Regulation of Macrophage Functions. Trends. Cell Biol..

[34] Infantino V., Iacobazzi V., Palmieri F., Menga A. (2013). ATP-Citrate Lyase Is Essential for Macrophage Inflammatory Response. Biochem. Biophys. Res. Commun..

[35] Horton J.D., Goldstein J.L., Brown M.S. (2002). SREBPs: Activators of the Complete Program of Cholesterol and Fatty Acid Synthesis in the Liver. J. Clin. Invest..

[36] Shimano H., Sato R. (2017). SREBP-Regulated Lipid Metabolism: Convergent Physiology—Divergent Pathophysiology. Nat. Rev. Endocrinol..

[37] Huang S.C.C., Everts B., Ivanova Y., O’sullivan D., Nascimento M., Smith A.M., Beatty W., Love-Gregory L., Lam W.Y., O’Neill C.M. (2014). Cell-Intrinsic Lysosomal Lipolysis is Essential for Alternative Activation of Macrophages. Nat. Immunol..

[38] Schlager S., Vujic N., Korbelius M., Duta-Mare M., Dorow J., Leopold C., Rainer S., Wegscheider M., Reicher H., Ceglarek U. (2017). Lysosomal Lipid Hydrolysis Provides Substrates for Lipid Mediator Synthesis in Murine Macrophages. Oncotarget.

[39] Zhang H. (2018). Lysosomal Acid Lipase and Lipid Metabolism: New Mechanisms, New Questions, and New Therapies. Curr. Opin. Lipidol..

[40] Rayner K.J., Suárez Y., Dávalos A., Parathath S., Fitzgerald M.L., Tamehiro N., Fisher E.A., Moore K.J., Fernández-Hernando C. (2010). MiR-33 Contributes to the Regulation of Cholesterol Homeostasis. Science.

[41] Najafi-Shoushtari S.H., Kristo F., Li Y., Shioda T., Cohen D.E., Gerszten R.E., Näär A.M. (2010). MicroRNA-33 and the SREBP Host Genes Cooperate to Control Cholesterol Homeostasis. Science.

[42] Houten S.M., Wanders R.J.A. (2010). A General Introduction to the Biochemistry of Mitochondrial Fatty Acid β-Oxidation. J. Inherit. Metab. Dis..

[43] Thiam A., Farese R., Walther T. (2013). The Biophysics and Cell Biology of Lipid Droplets. Nat. Rev. Mol. Cell Biol..

[44] JCI—Regulation and Mechanisms of Macrophage Cholesterol Efflux. https://www.jci.org/articles/view/16391.

[45] Zhu X., Lee J.-Y., Timmins J.M., Brown J.M., Boudyguina E., Mulya A., Gebre A.K., Willingham M.C., Hiltbold E.M., Mishra N. (2008). Increased Cellular Free Cholesterol in Macrophage-Specific Abca1 Knock-out Mice Enhances pro-Inflammatory Response of Macrophages. J. Biol. Chem..

[46] Hsieh W.-Y., Zhou Q.D., York A.G., Williams K.J., Scumpia P.O., Kronenberger E.B., Hoi X.P., Su B., Chi X., Bui V.L. (2020). Toll-Like Receptors Induce Signal-Specific Reprogramming of the Macrophage Lipidome. Cell Metab..

[47] Oishi Y., Spann N.J., Link V.M., Muse E.D., Strid T., Edillor C., Kolar M.J., Matsuzaka T., Hayakawa S., Tao J. (2017). SREBP1 Contributes to Resolution of Pro-Inflammatory TLR4 Signaling by Reprogramming Fatty Acid Metabolism. Cell Metab..

[48] Blanc M., Hsieh W.Y., Robertson K.A., Watterson S., Shui G., Lacaze P., Khondoker M., Dickinson P., Sing G., Rodríguez-Martín S. (2011). Host Defense against Viral Infection Involves Interferon Mediated Down-Regulation of Sterol Biosynthesis. PLoS Biol..

[49] Funk C.D. (2001). Prostaglandins and Leukotrienes: Advances in Eicosanoid Biology. Science.

[50] van der Laan L.J., Döpp E.A., Haworth R., Pikkarainen T., Kangas M., Elomaa O., Dijkstra C.D., Gordon S., Tryggvason K., Kraal G. (1999). Regulation and Functional Involvement of Macrophage Scavenger Receptor MARCO in Clearance of Bacteria In Vivo. J. Immunol..

[51] Palecanda A., Paulauskis J., Al-Mutairi E., Imrich A., Qin G., Suzuki H., Kodama T., Tryggvason K., Koziel H., Kobzik L. (1999). Role of the Scavenger Receptor MARCO in Alveolar Macrophage Binding of Unopsonized Environmental Particles. J. Exp. Med..

[52] The Scavenger Receptor MARCO Modulates TLR-Induced Responses in Dendritic Cells | PLoS ONE. https://journals.plos.org/plosone/article?id=10.1371/journal.pone.0104148.

[53] Józefowski S., Kobzik L. (2004). Scavenger Receptor A Mediates H_2_O_2_ Production and Suppression of IL-12 Release in Murine Macrophages. J. Leukoc. Biol..

[54] Dorrington M.G., Roche A.M., Chauvin S.E., Tu Z., Mossman K.L., Weiser J.N., Bowdish D.M.E. (2013). MARCO Is Required for TLR2- and Nod2-Mediated Responses to Streptococcus Pneumoniae and Clearance of Pneumococcal Colonization in the Murine Nasopharynx. J. Immunol..

[55] Su P., Wang Q., Bi E., Ma X., Liu L., Yang M., Qian J., Yi Q. (2020). Enhanced Lipid Accumulation and Metabolism Are Required for the Differentiation and Activation of Tumor-Associated Macrophages. Cancer. Res..

[56] Eisinger S., Sarhan D., Boura V.F., Ibarlucea-Benitez I., Tyystjärvi S., Oliynyk G., Arsenian-Henriksson M., Lane D., Wikström S.L., Kiessling R. (2020). Targeting a Scavenger Receptor on Tumor-Associated Macrophages Activates Tumor Cell Killing by Natural Killer Cells. Proc. Natl. Acad. Sci. USA.

[57] La Fleur L., Botling J., He F., Pelicano C., Zhou C., He C., Palano G., Mezheyeuski A., Micke P., Ravetch J.V. (2021). Targeting MARCO and IL37R on Immunosuppressive Macrophages in Lung Cancer Blocks Regulatory T Cells and Supports Cytotoxic Lymphocyte Function. Cancer Res..

[58] Dong Y., D’Mello C., Pinsky W., Lozinski B.M., Kaushik D.K., Ghorbani S., Moezzi D., Brown D., Melo F.C., Zandee S. (2021). Oxidized Phosphatidylcholines Found in Multiple Sclerosis Lesions Mediate Neurodegeneration and Are Neutralized by Microglia. Nat. Neurosci..

[59] Sun X., Seidman J.S., Zhao P., Troutman T.D., Spann N.J., Que X., Zhou F., Liao Z., Pasillas M., Yang X. (2020). Neutralization of Oxidized Phospholipids Ameliorates Non-Alcoholic Steatohepatitis. Cell Metab..

[60] Serbulea V., Upchurch C.M., Schappe M.S., Voigt P., DeWeese D.E., Desai B.N., Meher A.K., Leitinger N. (2018). Macrophage Phenotype and Bioenergetics Are Controlled by Oxidized Phospholipids Identified in Lean and Obese Adipose Tissue. Proc. Natl. Acad. Sci. USA.

[61] Jin H.-R., Wang J., Wang Z.-J., Xi M.-J., Xia B.-H., Deng K., Yang J.-L. (2023). Lipid Metabolic Reprogramming in Tumor Microenvironment: From Mechanisms to Therapeutics. J. Hematol. Oncol..

[62] FASTQC A Quality Control Tool for High Throughput Sequence Data | BibSonomy. https://www.bibsonomy.org/bibtex/f230a919c34360709aa298734d63dca3.

[63] Ewels P., Magnusson M., Lundin S., Käller M. (2016). MultiQC: Summarize Analysis Results for Multiple Tools and Samples in a Single Report. Bioinformatics.

[64] Martin M. (2011). Cutadapt Removes Adapter Sequences from High-Throughput Sequencing Reads. EMBnet. J..

[65] Dobin A., Davis C.A., Schlesinger F., Drenkow J., Zaleski C., Jha S., Batut P., Chaisson M., Gingeras T.R. (2013). STAR: Ultrafast Universal RNA-Seq Aligner. Bioinformatics.

[66] Trapnell C., Williams B.A., Pertea G., Mortazavi A., Kwan G., van Baren M.J., Salzberg S.L., Wold B.J., Pachter L. (2010). Transcript Assembly and Quantification by RNA-Seq Reveals Unannotated Transcripts and Isoform Switching during Cell Differentiation. Nat. Biotechnol..

[67] Risso D., Ngai J., Speed T.P., Dudoit S. (2014). Normalization of RNA-Seq Data Using Factor Analysis of Control Genes or Samples. Nat. Biotechnol..

[68] Langfelder P., Horvath S. (2008). WGCNA: An R Package for Weighted Correlation Network Analysis. BMC Bioinform..

[69] Wu T., Hu E., Xu S., Chen M., Guo P., Dai Z., Feng T., Zhou L., Tang W., Zhan L. (2021). clusterProfiler 4.0: A Universal Enrichment Tool for Interpreting Omics Data. Innovation.

[70] Szklarczyk D., Gable A.L., Nastou K.C., Lyon D., Kirsch R., Pyysalo S., Doncheva N.T., Legeay M., Fang T., Bork P. (2021). The STRING Database in 2021: Customizable Protein–Protein Networks, and Functional Characterization of User-Uploaded Gene/Measurement Sets. Nucleic Acids Res..

[71] Csardi G., Nepusz T. (2005). The Igraph Software Package for Complex Network Research. InterJournal Complex Syst..

