# Supplementary material for: In Silico Analysis Revealed Marco (SR-A6) and Abca1/2 as Potential Regulators of Lipid Metabolism in M1 Macrophage Hysteresis
Source: Int J Mol Sci. 2024 Dec 26;26(1):111. doi: 10.3390/ijms26010111 (PMC11719740; doi:10.3390/ijms26010111)
Supplement: Supplementary file 1 [file ijms-26-00111-s001.zip › Supplementary Figures S1-S9.pdf]

Supplementary Materials

Supplementary Figures

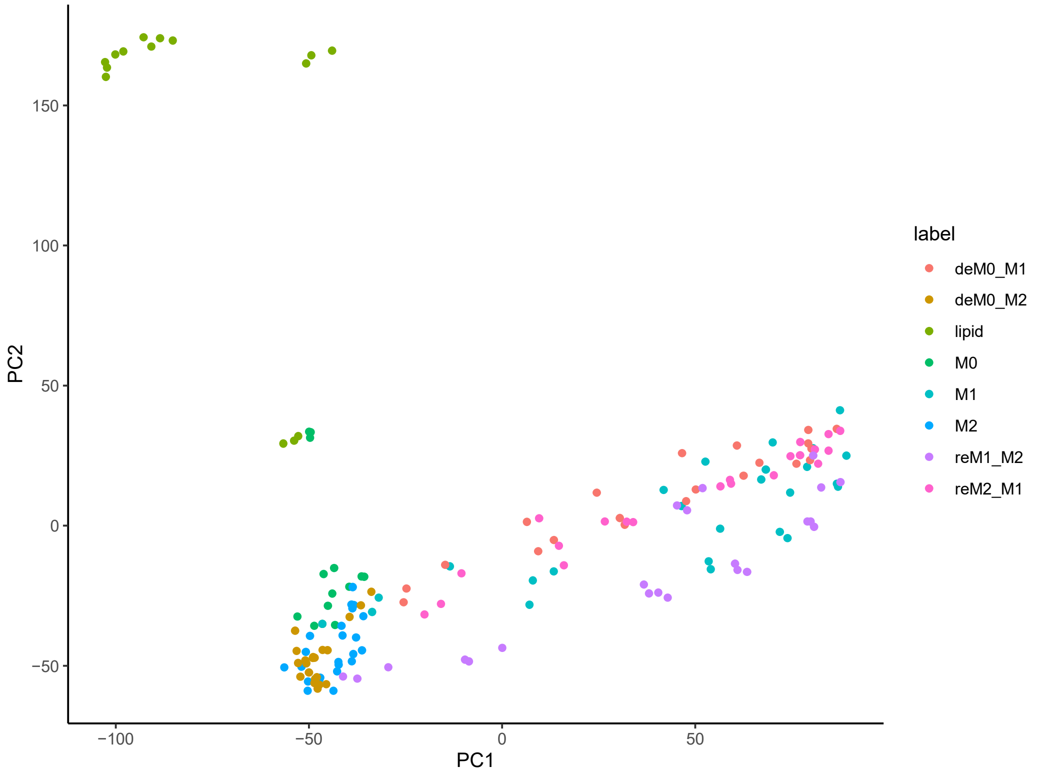

Figure S1. PCA of RNA-seq data prior to batch effect correction. The dot colors represent different macrophage phenotypes and lipid-loaded macrophages, as illustrated in the figure.

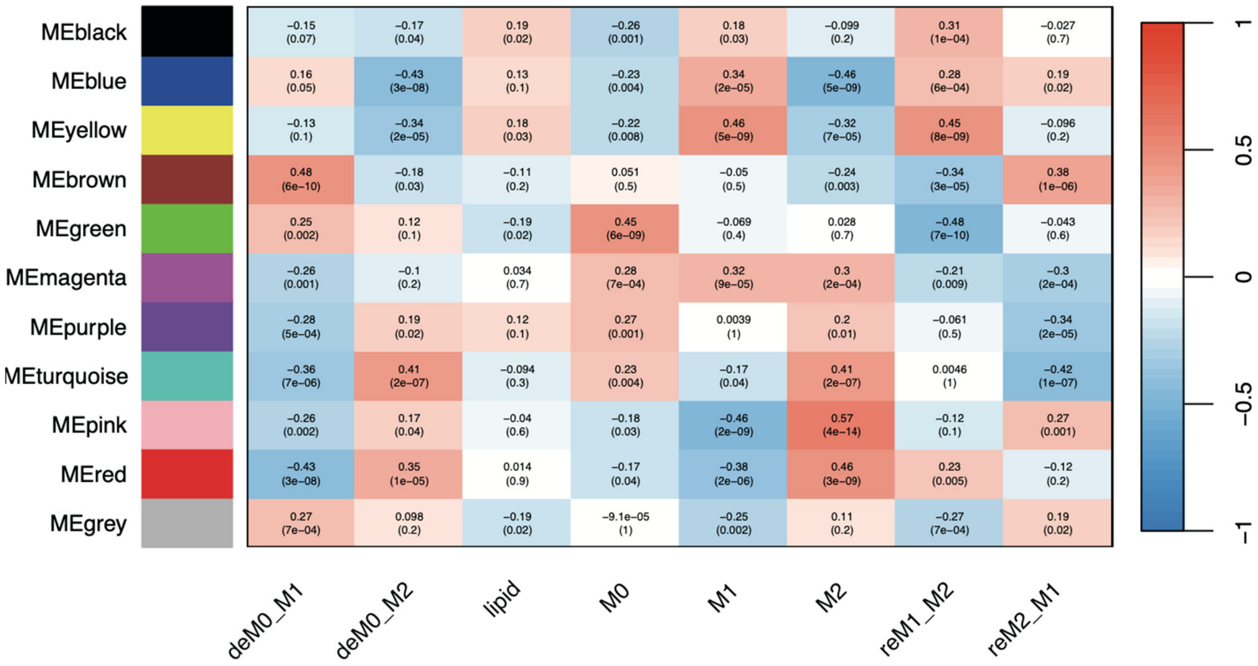

Figure S2. Preliminary WGCNA results using lipid-loaded and polarized samples. Heatmap showing the associations between module eigengenes, and all identified co-expression modules.

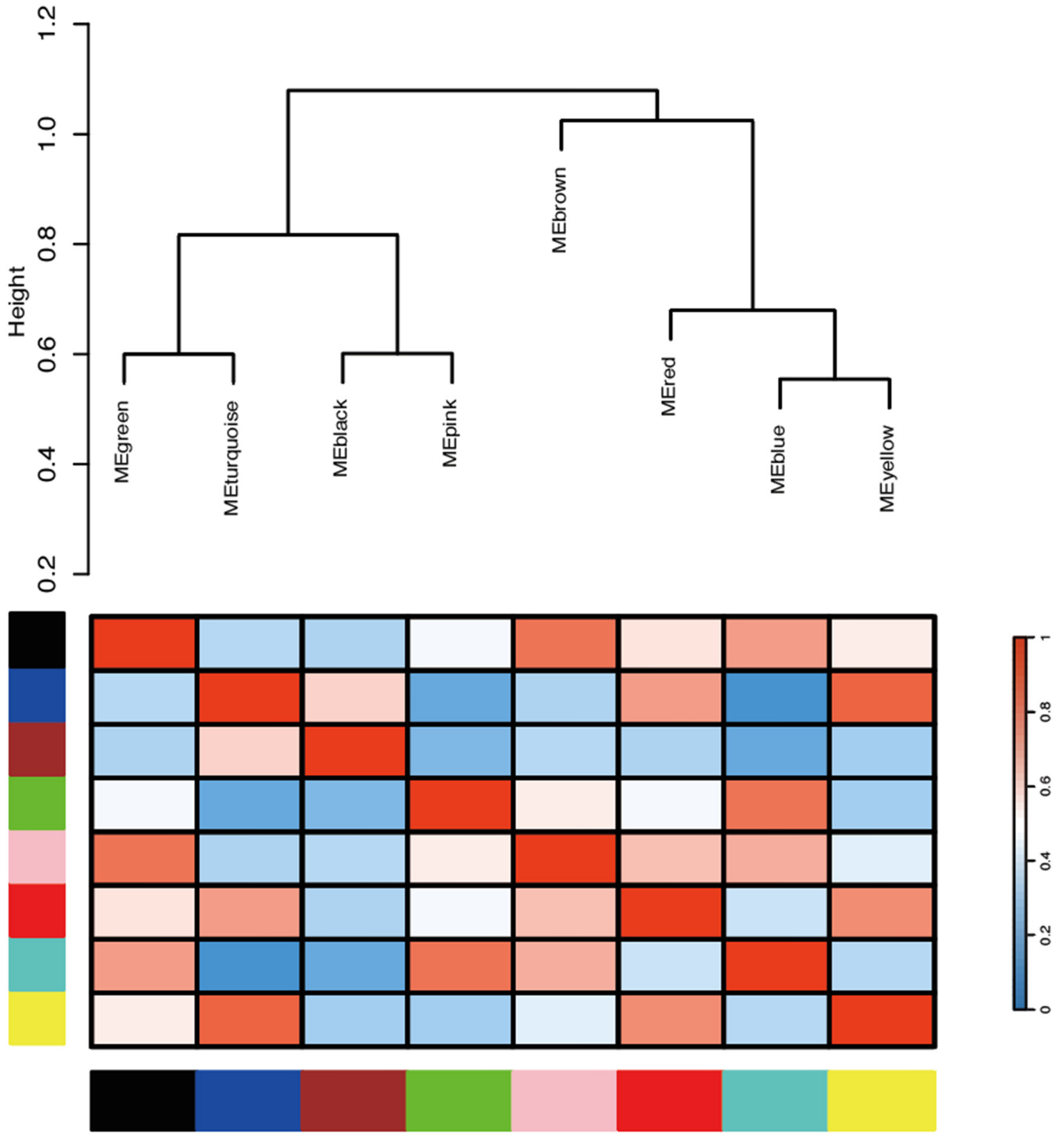

Figure S3. The eigengene network's heatmap shows correlations between various modules; those with strong connections are grouped together.

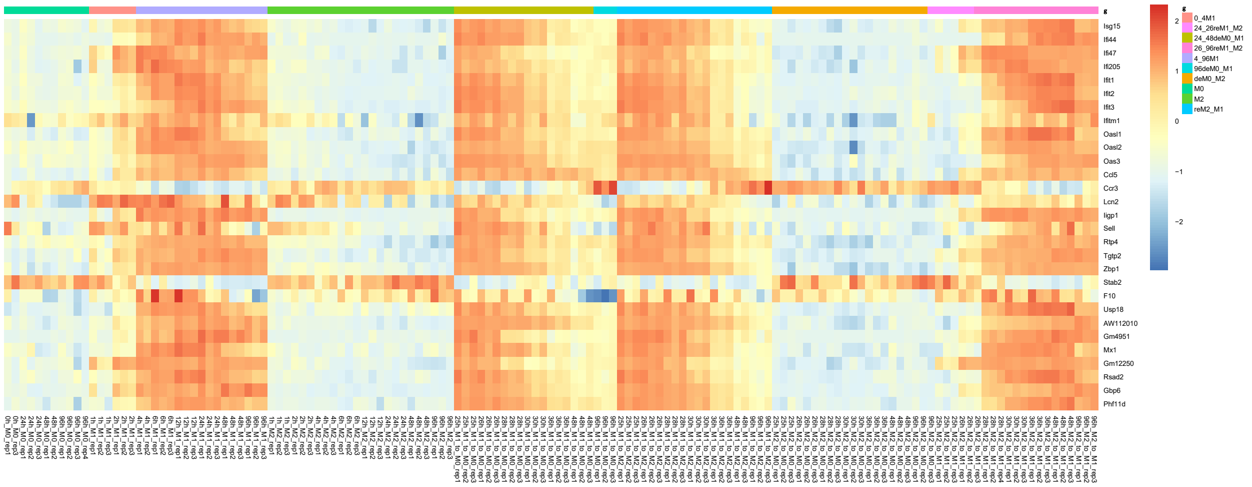

Figure S4. The gene expression levels of hysteresis genes in the MEblue across macrophage polarization data.

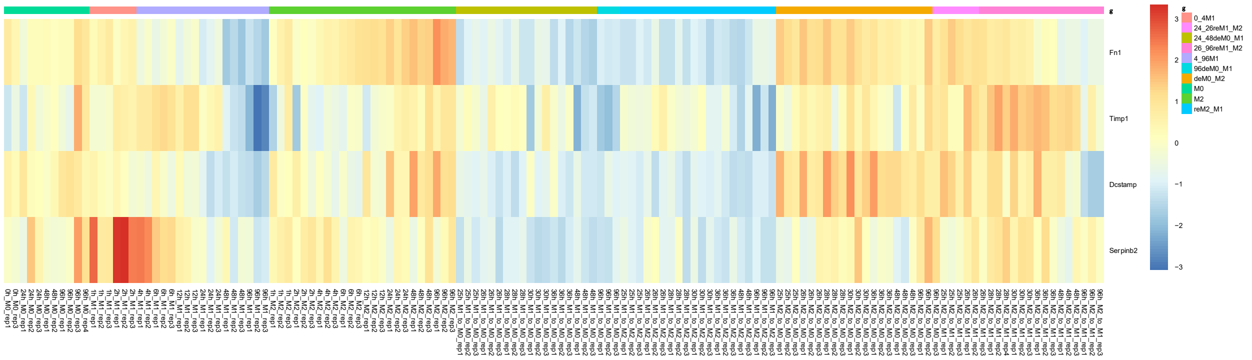

Figure S5. The gene expression levels of hysteresis genes in the MEbrown across macrophage polarization data.

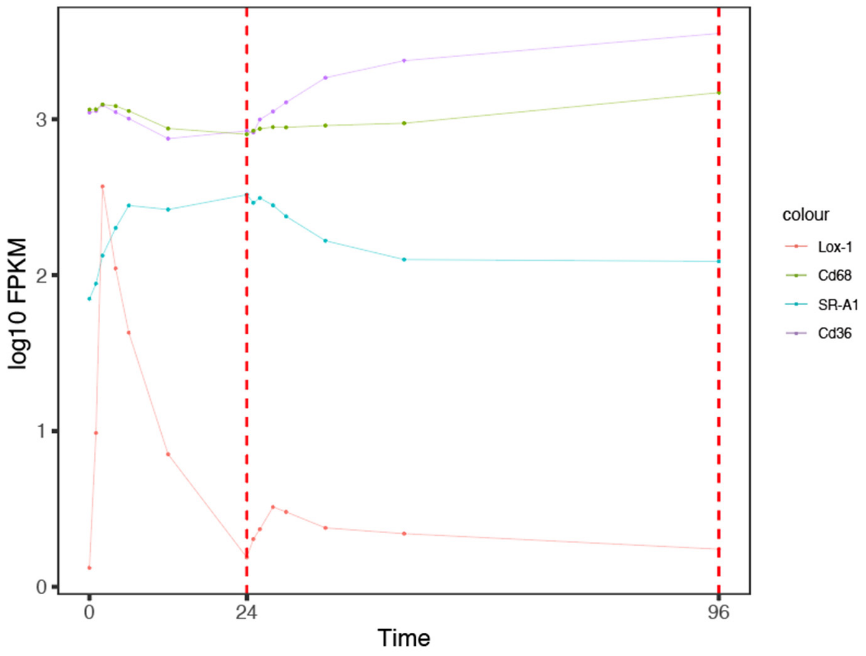

Figure S6. Gene expression levels of Lox-1, Cd68, SR-A1, Cd36 under M0->M1->M2 between 0 to 96 hours.

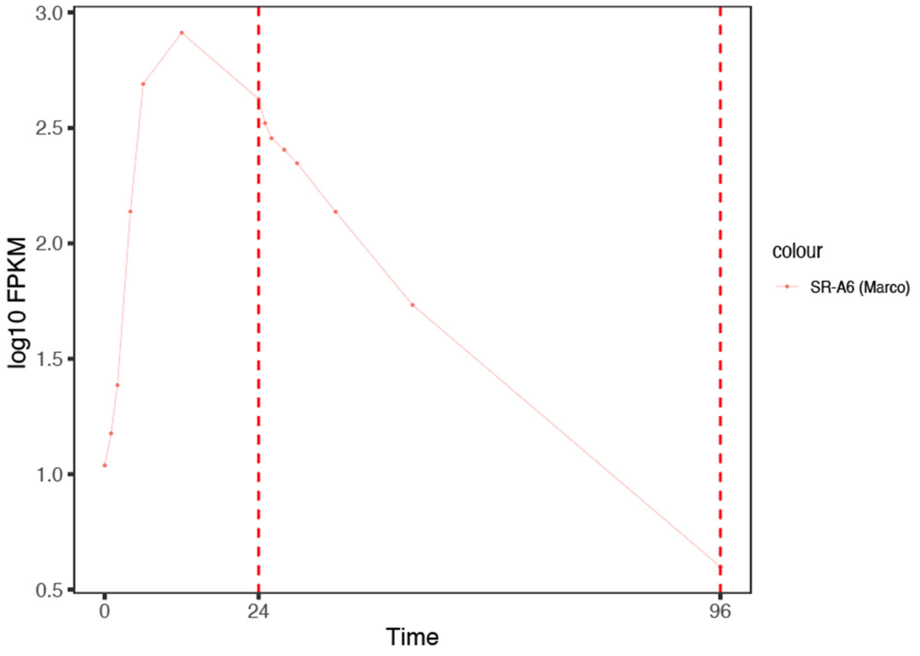

Figure S7. Gene expression levels of Marco under M0->M1->M2 between 0 to 96 hours.

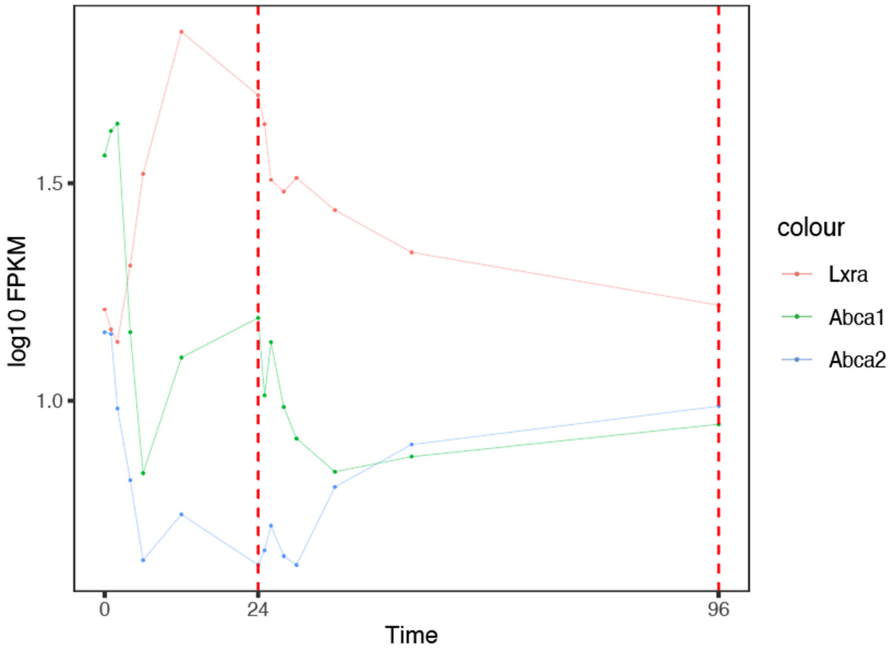

Figure S8. Gene expression levels of Lxra, Abca1, Abca2 under M0->M1->M2 between 0 to 96 hours.

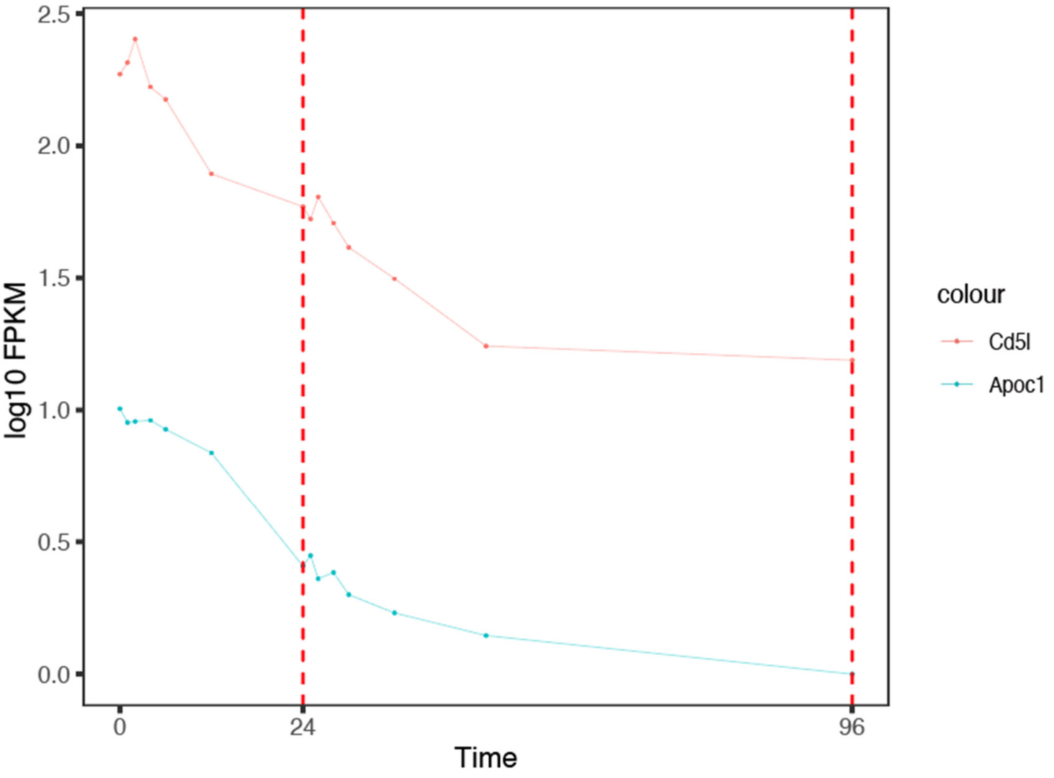

Figure S9. Gene expression levels of Cd5l, Apoc1under M0->M1->M2 between 0 to 96 hours.
